# Cantharidin: a double-edged sword in medicine and toxicology

**DOI:** 10.3389/fphar.2025.1644186

**Published:** 2025-09-17

**Authors:** Jie Zhang, Tongtong Tian, Canyu Li, Yong Liu, Yunyun Wang, Ling Liu, Liang Liu, Yufeng Yao

**Affiliations:** ^1^ Department of Forensic Medicine, Tongji Medical College, Huazhong University of Science and Technology, Wuhan, Hubei, China; ^2^ Key Laboratory of Molecular Biophysics of the Ministry of Education, College of Life Science and Technology and Center for Human Genome Research, Huazhong University of Science and Technology, Wuhan, Hubei, China; ^3^ Prenatal Diagnosis Center, The Third Affiliated Hospital of Zhengzhou University, Zhengzhou, Henan, China

**Keywords:** anti-cancer drug, cantharidin, pharmacology, toxicology, analogues

## Abstract

Cantharidin (CTD), a natural terpenoid toxin secreted by blister beetles, acts as a potent inhibitor of protein phosphatase. As the principal active component of Mylabris, a traditional Chinese medicine, CTD has attracted considerable interest due to its dual properties, combining potent anti-tumor activity with significant toxicity. Contemporary pharmacological research demonstrates that CTD inhibits the growth and proliferation of diverse cancer cells lines. It exhibits antibacterial and antiparasitic properties, and demonstrates pesticidal activity in agricultural applications. Despite these benefits, CTD exhibits a prominent double-edged profile, marked by severe toxic effects, including cardiotoxicity, nephrotoxicity, gastrointestinal toxicity, and reproductive toxicity. Our prior research has identified the heart and liver as primary targets of CTD’s acute toxicity, where it induces apoptosis and necrosis of cardiomyocytes and hepatocytes. Recent efforts to mitigate its toxicity while preserving efficacy have focused on the structural modifications of CTD and the development of its derivatives. Additionally, CTD has been demonstrated to enhance anti-tumor efficacy when combined with other drugs, particularly against certain drug-resistant tumors. This review comprehensively evaluates CTD’s pharmacology and toxicology, synthesizes pertinent toxicological data, and explores strategies for toxicity reduction to guide future research.

## 1 Introduction

**FIGURE 1:**
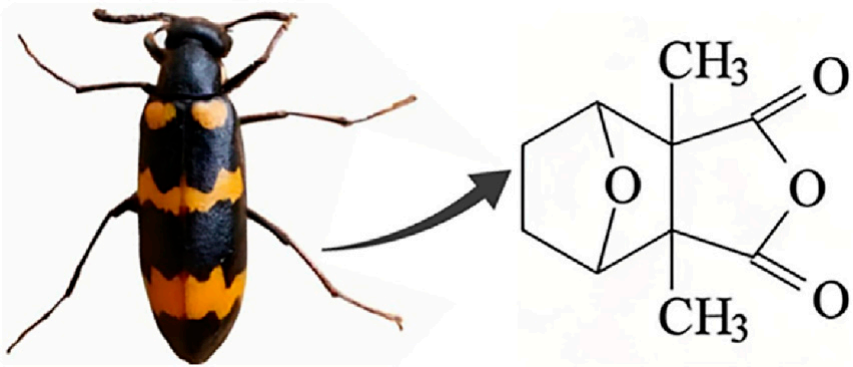
Chemical structure of CTD.

Natural products have the potential to yield significant advancements in disease treatment. Their bioactive constituents frequently contribute to unexpected therapeutic insights, particularly within the domain of traditional Chinese medicine (TCM). Cantharidin (CTD), a toxic tricyclic monoterpene, is a naturally occurring compound derived from blister beetles, notably as the primary active constituent present in bodies of southern meloid beetles, such as Mylabris phalerata or Mylabris variabilis, and Spanish flies (Lytta vesicatoria). Typically, species of the genus *Mylabris* contain 1%–1.5% CTD by weight (Nicholls et al., 1990). CTD serves as an effective defensive chemical, synthesized and secreted by male beetles from their legs (Carrel et al., 1993), exhibiting potent biological activity that protects them from predators. Its chemical formula is C_10_H_12_O_4_ (Figure 1), and its molecular structure is notably stable, retaining its toxicity even in the remains of deceased beetles over extended periods.

Owing to its distinctive pharmacological properties, CTD has been widely utilized globally across centuries. It is used to chiefly to treat conditions such as ulcers and lymphadenitis, acting as a topical vesicant that induces separation syndrome. Its clinical applications encompass wart removal, molluscum contagiosum (MC), and acquired perforating dermatosis, as well as the treatment of ulcers and leishmaniasis (Torbeck et al., 2014). In China, its use dates back approximately 2,000 years (Knapp et al., 1998), where it has been commonly applied to manage MC, rabies and induce abortion, though it was classified as highly toxic owing to its elevated mortality risk. In Europe and Africa, CTD was historically employed as an aphrodisiac, with oral administration triggering abnormal penile erection (Ellenhorn, 1997). Beyond these uses, CTD exhibits notable bactericidal and insecticidal properties, inhibiting the growth of vaginal trichomonas, nematodes, and ticks (Whitman et al., 2019). Its anticancer activity stems from potent inhibition of mammalian phosphatase 1 (PP1) and protein phosphatase 2A (PP2A) activities (Wang et al., 2018; Pan et al., 2019). Currently, CTD is predominantly utilized in anticancer therapies, bolstered by extensive *in vivo* and *in vitro* studies confirming its efficacy against liver, pancreatic, colon, bladder, and breast cancers, with additional anti-tumor effects documented in oral cancer and leukemia (Deng et al., 2013). Furthermore, CTD functions as an effective pesticide, demonstrating high toxicity to numerous insects, particularly Coleoptera (Khan et al., 2013).

Although CTD offers extensive clinical applications, its pronounced toxic effects restrict its widespread adoption. Exposure to high doses can induce severe reactions in both animals and humans. Symptoms of CTD poisoning include formation of large cutaneous blisters, hematuria, myocardial damage, mucosal erosion and bleeding in the upper gastrointestinal tract, gastrointestinal erosion, renal failure, and abnormal penile erection (Karras et al., 1996). Documented reports highlight fatalities resulting from its ingestion for dermatological conditions or abortion (Polettini et al., 1992), alongside cases of homicide (Chen and Huang, 2013). Despite this wide range of clinical symptoms, existing information on CTD toxicity remains markedly limited. This review provides a comprehensive evaluation of the characteristics of CTD, including its analogs, classification, clinical therapeutic mechanisms, and toxicity studies, with the aim of enhancing its clinical application while offering forensic professionals valuable insights and guidance for identifying cases of CTD poisoning. We aim to review the crucial pharmacological and toxicological mechanisms of CTD in the human body. This will enhance our knowledge of its toxic effects on body organs, clarify its potential therapeutic applications, and provide forensic professionals with valuable insights for identifying CTD poisoning cases. Moreover, we evaluated the modified products of CTD to advance our knowledge of CTD, which leads to better management of the poisonings.

## 2 Pharmacological effects

### 2.1 Anticancer effects

#### 2.1.1 CTD induces DNA damage and inhibits cancer cell growth and proliferation

**FIGURE 2:**
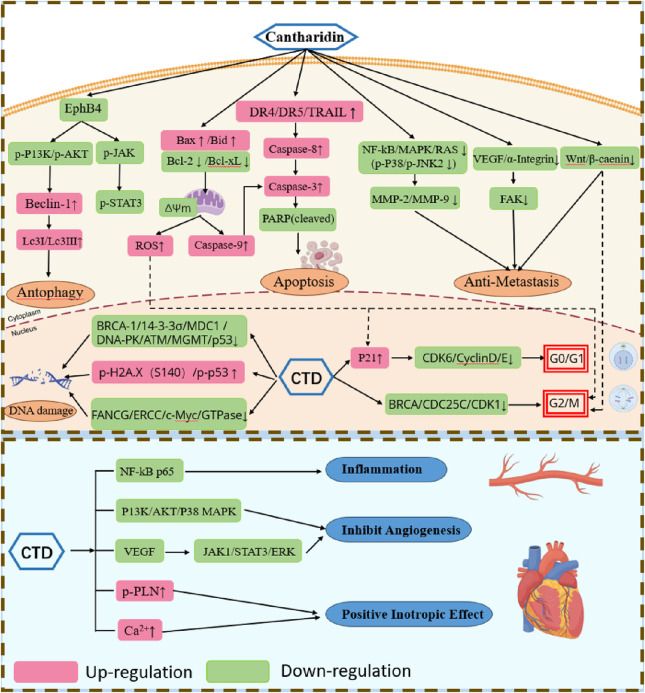
Pharmacological actions of CTD in anticancer and cardiovascular effects.

CTD exhibits potent cytotoxicity, inducing DNA damage in cancer cells by compromising the integrity of their genomes, leading to DNA fragmentation (Li et al., 2017), inhibition of DNA repair and damage, and subsequent suppression of cancer cell growth and proliferation (Figure 2). Its cytotoxicity has been confirmed across numerous previous studies (Naz et al., 2020), spanning leukemia cell line (CCRF-CEM), skin cancer (A431), gastric cancer (BGC823) breast cancer cell (MCF-7), and hepatocellular carcinoma (HepG2), as comprehensively reported in the literature. CTD-induced comet tails and DNA condensation have also been observed in human lung cancer (NCI-H460) cells and bladder cancer cells (Kuo et al., 2015; Hsia et al., 2015). Moreover, CTD emerges as a promising alternative therapy for drug-resistant cancer cells. For instance, it strongly inhibits the growth of imatinib-resistant chronic myeloid leukemia (CML) cells, induces DNA damage in these cells, and reduces BCR-ABL transcription levels, thereby curtailing malignant proliferation and transformation without harming normal blood cells (Sun et al., 2016). Studies (Zheng et al., 2008) by Zheng et al. demonstrate that CTD reverses multidrug resistance in human hepatocellular carcinoma HepG2/ADM cells by suppressing P-glycoprotein mRNA transcription and the protein expression. Beyond direct DNA damages, CTD also significantly disrupts genes and proteins critical to DNA repair in cancer cells. Research by Meng-Dan Xu et al. reveals that CTD enhances DNA damage and inhibits DNA repair-related genes via JNK, ERK, p38, PKC, and NF-κB pathways, thereby sensitizing pancreatic cancer cells to radiotherapy (Xu et al., 2018). In HL-60 acute myeloid leukemia cells, CTD treatment not only decreases expression of genes encoding proteins involved in DNA replication (e.g., DNA polymerase δ), DNA repair (e.g., FANCG, ERCC), and energy metabolism (e.g., isocitrate dehydrogenase α, ADP/ATP translocase), but also decreases the expression of genes encoding proteins with oncogenic activity (e.g., c-Myc, GTPases) and genes exhibiting tumor-specific expression (e.g., phosphoinositide 3-kinase) (Zhang et al., 2004). These findings indicate that CTD effectively cause DNA damage in cancer cells and inhibit their proliferation, offering broad therapeutic potential for managing drug-resistant cancer cells.

#### 2.1.2 Induction of apoptosis in cancer cells

CTD induces apoptosis in cancer cells via both intrinsic and extrinsic pathways (Figure 2). It activates mitochondrial-dependent intrinsic pathways and stimulates of extracellular ligands to engage death receptors (Pistritto et al., 2016). Within the intrinsic pathway, the B-cell lymphoma 2 (Bcl-2) protein plays a pivotal role in regulating mitochondrial outer membrane permeabilization. In the human lung cancer cell line A540, CTD induces apoptosis by increasing levels of Bax protein and active caspase-3, while reducing Bcl-2 translation (Liu et al., 2018). It also enhances the production of reactive oxygen species (ROS) and Ca^2+^, reduces mitochondrial transmembrane potential, upregulates expression of Caspase-3, Caspase-8, Cytochrome C, and Bax, while downregulating Bcl-xL levels (Hsia et al., 2015). Similar expression profiles have been observed in studies of CTD on breast cancer cells, squamous cell carcinoma of the tongue, and bladder cancer cells (Kuo et al., 2015; Tian et al., 2015). Furthermore, CTD suppresses hepatocellular carcinoma by regulating the JAK2/STAT3 and PI3K/Akt pathways in an EphB4-dependent manner (Zhu et al., 2020). The extrinsic pathway of apoptosis is mediated by extracellular ligands binding to their corresponding death receptors. Multiple studies (Li et al., 2017; Li et al., 2011) report significant alterations in the expression of death receptors (including DR4, DR5, and TRAIL) and other mediators of the extrinsic pathway. For instance, CTD exerts its anti-tumor effect on human pancreatic cancer cells via the extrinsic apoptosis pathway by elevating levels of TNF-α, TRAIL-R1, and TRAIL-R2. These alterations promote apoptosis in these cancer cells through the activation of caspases and subsequent cell death.

#### 2.1.3 Induction of cell cycle arrest in cancer cells

In higher organisms, the cell cycle is a tightly regulated process, divided into four phases: G1, S, G2, and M phase. In higher organisms, the cell cycle is a highly regulated event controlled by various mechanisms. Treatment of chronic myelogenous leukemia (CML) cells K562 cells and imatinib-resistant K562R cells with CTD induces mitotic arrest, mediated by activation of the cyclin B1/Cdc2 complex and downregulation of cyclin D1. After 24 h of treatment, 19.2%–24.5% of K562 cells and 10.8%–13.0% of K562R cells are arrested in mitosis phase (Sun et al., 2016). CTD induces G0/G1 phase arrest in human bladder cancer cells and human skin cancer cells, correlating with decreased expression of cyclin D, E, and CDK6 proteins (Li et al., 2017; Kuo et al., 2015). In HepG2 hepatocellular carcinoma cells, CTD treatment significantly increased the cell population in the G2/M phase and decreased the cell population in the G1 phase (Le et al., 2016). Similarly, in pancreatic cancer and osteosarcoma cells, CDK1 activity is suppressed, thereby inducing G2/M phase arrest (Li et al., 2011; Chen et al., 2019). Beyond cell cycle arrest in liver cancer cells, CTD modulates immune responses by regulating expression of chemokine-related gene, inflammatory cytokines, and immune checkpoint genes, thereby inhibiting immune cell infiltration to suppress tumorigenesis (Yan et al., 2023). Collectively, CTD modulates a range of cycle-related proteins to arrest cancer cells at distinct phases of the cell cycle. In general, CTD halts mitosis in cancer cells by targeting key cycle-related proteins, while cancer cells post-division may be arrested in the G0 phase to prevent further division activities.

#### 2.1.4 Inhibition of cancer cell metastasis

Cancer cells degrade the extracellular matrix (ECM) to invade normal tissues, With matrix metalloproteinases (MMPs) playing a pivotal role in this ECM degradation. Of the over 20 MMPs identified to date, MMP-2 and MMP-9 are intrinsic factors in cancer metastasis (Kim et al., 2013). CTD prevents adhesion, migration, and invasion of various cancer cells through multiple signaling pathways (Figure 2). For instance, it downregulates MMP-2 and MMP-9 mRNA expression in bladder cancer cells by modulating the p38 and JNK1/2, MAPK signaling pathways (Huang et al., 2013), inhibits the PI3K/AKT pathway in gastric cancer cells (Song et al., 2020), and reduces expression of NF-κB, p65, and AKT to block the MAPK pathway in NCI-H460 and A375 cells (Ji et al., 2015; Hsia et al., 2016). Additionally, in MCF-7 cells, CTD suppresses both growth and adhesion by downregulating α2 integrin via the protein kinase C pathway (Shou et al., 2013). In pancreatic cancer cells, CTD diminishes proliferation and migration by targeting the Wnt/β-catenin signaling pathway through β-catenin inhibition via β-TrCP proteins (Wu et al., 2014). Similarly, in osteosarcoma cells, CTD inhibits proliferation and migration by downregulating miR-214-3p, thereby upregulating DKK3 and reducing β-catenin nuclear translocation (Hu et al., 2021). Although aerobic glycolysis’s role in cancer growth and metastasis remains poorly understood, CTD inhibits pyruvate kinase M2 (PKM2) nuclear translocation and disrupts the GLUT1/PKM2 glycolytic loop, promoting a shift from aerobic glycolysis to oxidation and subsequently reversing breast cancer metastasis (Pan et al., 2019). Collectively, CTD suppresses cancer cell metastasis and invasion through diverse pathways, positioning it as a promising therapeutic candidate for further development.

### 2.2 Cardiovascular effects

Serine/threonine phosphatases, including PP1, PP2A, and PP2B, account for approximately 90% of the dephosphorylation events in the heart. The pronounced toxicity of CTD and its analogs in mammals stems from their elevated affinity and specificity for CTD-binding proteins, identified as PP1 and PP2A, and the efficiency of CTD in inhibiting PP2A is 10 times that of inhibiting PP1 (Neumann et al., 1995). CTD enhances myocardium contractility by regulating the phosphorylation state of proteins, specifically by inhibiting cardiac protein phosphatases, which leads to sustained phosphorylation of PLN (phospholamban). Additionally, it can moderately increase the L-type calcium channel current in guinea-pig cardiomyocytes, thereby exerting positive inotropic effects without increasing cAMP (cyclic adenosine monophosphate) content, distinguishing it from the positive inotropic mode of adrenaline (Neumann et al., 1995). However, this effect is not entirely attributable to calcium ions, as an increase in calcium ions without CTD at the same concentration does not yield equivalent contractile force, suggesting that CTD may increase myocardial contractility through pathways other than calcium current inhibition (Knapp et al., 1998; Neumann et al., 1995). PP2A activity is linked to the activities of a number of key ion channels, including the L-type calcium channel (Cav1.2), ryanodine receptor 2 (RyR2), Na^+^/Ca^2+^ exchanger (NCX), and Na^+^/K^+^ ATPase (El Refaey et al., 2019). CTD inhibits PP2A activity, potentially influencing the above - mentioned receptors. CTD can inhibit the negative inotropic effects induced by various drugs in the human or animal heart, such as the negative inotropic effect caused by carbachol in the isolated human atrium and endothelin-1 in canine ventricular myocardium (Schwarz et al., 2024; Chu et al., 2003).

Moreover, CTD protects heart from ischemia and reduces infarct size through ERK phosphorylation dependency (Yang et al., 2011). However, it fails to alleviate reperfusion-induced damage. CTD can also enhance the contractility of smooth muscle, primarily by inhibiting phosphatase activity in aortic smooth muscle (Knapp et al., 1997) and increasing the phosphorylation state of vascular regulatory proteins. In contrast to its cardiac effects, CTD does not affect calcium homeostasis (Knapp et al., 1998), instead predominantly modulating vascular contraction and regulates the phosphorylation of myosin light chains. In addition, CTD influences coronary artery perfusion while exerting positive inotropic effects, potentially exerting contradictory cardiac impacts (Knapp et al., 1998). In vascular smooth muscle cells, CTD reduces proliferation and migration capabilities by inhibiting inflammatory responses, suggesting its potential application in post-angioplasty neointimal hyperplasia and restenosis (Qiu et al., 2019). Additionally, CTD inhibits vascular endothelial growth factor (VEGF)-induced JAK1/STAT3, ERK, and AKT signaling pathways, suggesting its potential for anti-angiogenic therapy (Wang T. et al., 2015).

### 2.3 Antimicrobial and insecticidal effects

**FIGURE 3:**
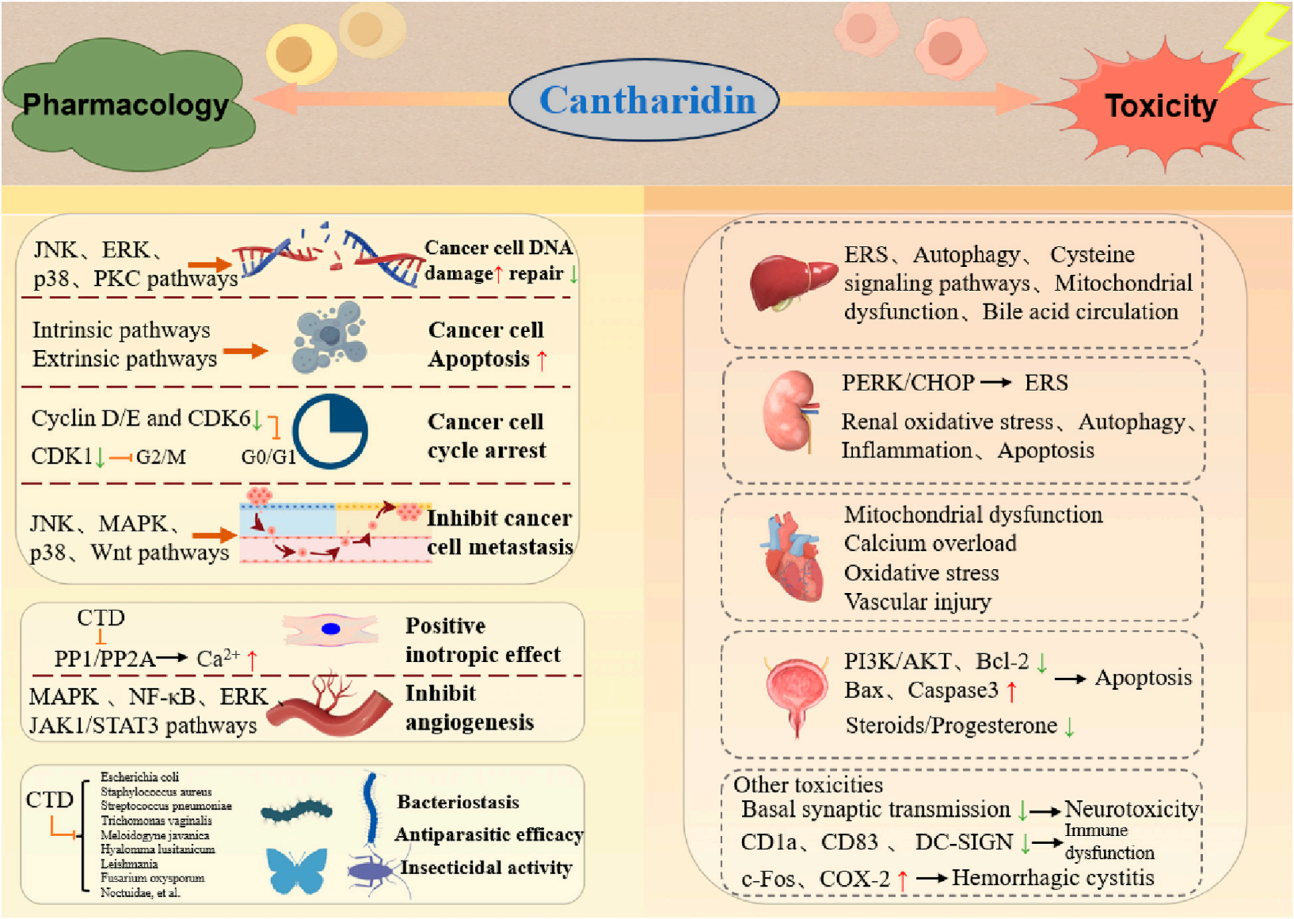
Double-edged profile of CTD in medicine and toxicology.

Despite its toxicity to many animals, some species such as the *Otis tarda* consume blister beetles to treat their own bacterial and parasite infections (Bravo et al., 2014). Experimental evidence demonstrates that CTD exerts concentration-dependent inhibitory effects on bacteria such as *Escherichia coli*, *Staphylococcus aureus*, and *Streptococcus pneumoniae*. Douglas et al. primarily studied by testing the antiparasitic efficacy of both pure CTD and extracts of *B. majalis* beetles against protozoa (*Trichomonas vaginalis*), a nematode (*Meloidogyne javanica*), and a tick (*Hyalomma lusitanicum*) (Whitman et al., 2019). Previously, CTD has shown good effects against severe *Leishmania* major infections (Ghaffarifar, 2010), achieving 80% growth inhibition *in vitro* at a concentration of 50 μg/mL, as well as efficacy *in vivo* in experimentally infected BALB/c mice. CTD also exerts a potent toxic effect on certain pests. Hong Sun et al. had shown that CTD can control *Periplaneta americana* (L.) through Serine/Threonine Protein Phosphatase Type 5 (Sun et al., 2020). In agriculture, CTD exhibits potent toxicity against lepidopteran pests (Noctuidae), showing low susceptibility to resistance, thus offering promise for agricultural applications (Li Y. et al., 2021). Additionally, CTD has been reported to possess insecticidal activity against pests such as Plutella xylostella, Helicoverpa armigera, Walker, Fabricius, and *Musca domestica* (Sun et al., 2022). Collectively, CTD displays activity against a wide range of organisms, including protozoa, nematodes, ticks, and insects (Figure 3). CTD shows great potential in the fields of animal husbandry and agriculture. In the animal field, considering its antibacterial and antiparasitic properties, it may be used in preventing and treating various animal diseases caused by bacteria or parasites. For example, it could be developed into veterinary drugs to protect the health of livestock and poultry, improve their survival rate and productivity. In the agricultural field, its pest-killing characteristic makes it a potential natural pesticide. It can be used to control agricultural pests, reduce the damage of pests to crops, and help increase crop yields. Moreover, compared with some traditional chemical pesticides, CTD is more environmentally friendly, which is in line with the concept of sustainable agricultural development.

### 2.4 Clinical applications of CTD

In July 2023, YCANTH™ (CTD 0.7% topical solution) was approved in the United States for the topical treatment of molluscum contagiosum in adult and pediatric patients aged 2 years and older (Keam, 2024). Mechanistically, CTD is absorbed by lipids in keratinocytes, where it activates neutral serine proteases, triggering progressive degeneration of desmosomal dense plaques. This results in selective acantholysis occurring intraepidermally, which heals over time without scarring. Blisters form within 24–48 h and resolve within 4–7 days (Torbeck et al., 2014). Additionally, CTD has been employed as an inflammatory model and in cancer treatment (Naz et al., 2020; Dinh et al., 2011). Sun et al. also reported that carboxylesterases (CarEs), glutathione S-transferases (GSTs), and cytochrome P450 monooxygenases (P450s) act as detoxifying enzymes for CTD, with P450 exhibiting consistent activity recovery comparable to that of phosphatases (Sun et al., 2022). Future research may explore incorporating detoxifying enzymes into treatment protocols to maximize the clinical utility of CTD. By screening for CTD-detoxifying enzymes, genetic testing can be performed on patients prior to clinical administration, aiming to provide guidance for determining clinical drug dosages.

In China, Aidi Injection (Z52020236, approved by the National Medical Products Administration), comprising extracts from Astragalus membranaceus, Acanthopanax senticosus, Panax ginseng C. A. Mey, and CTD from Lytta vesicatoria, has emerged as a widely utilized adjuvant chemotherapy agent for anti-tumor therapy (Zhang et al., 2025). Clinically, it is predominantly employed to treat malignant tumors, including hepatocellular carcinoma, lung cancer, bladder cancer, and colorectal cancer. It is typically administered in combination with other anti-tumor drugs for cancer treatment. For example, when combined with paclitaxel to treat advanced non-small cell lung cancer (NSCLC), its efficacy is comparable to that of the chemotherapy drug cisplatin, yet it exhibits reduced side effects (Xiao et al., 2019). The available evidence (Xiao et al., 2017) indicates that Aidi injection plus gemcitabine and cisplatin (GP) can significantly increase the clinical efficacy and improve the quality of life for patients (QOL) of patients with NSCLC. Meanwhile, CTD is also used to treat certain inflammatory diseases and gout.

## 3 Side effects of CTD

**FIGURE 4:**
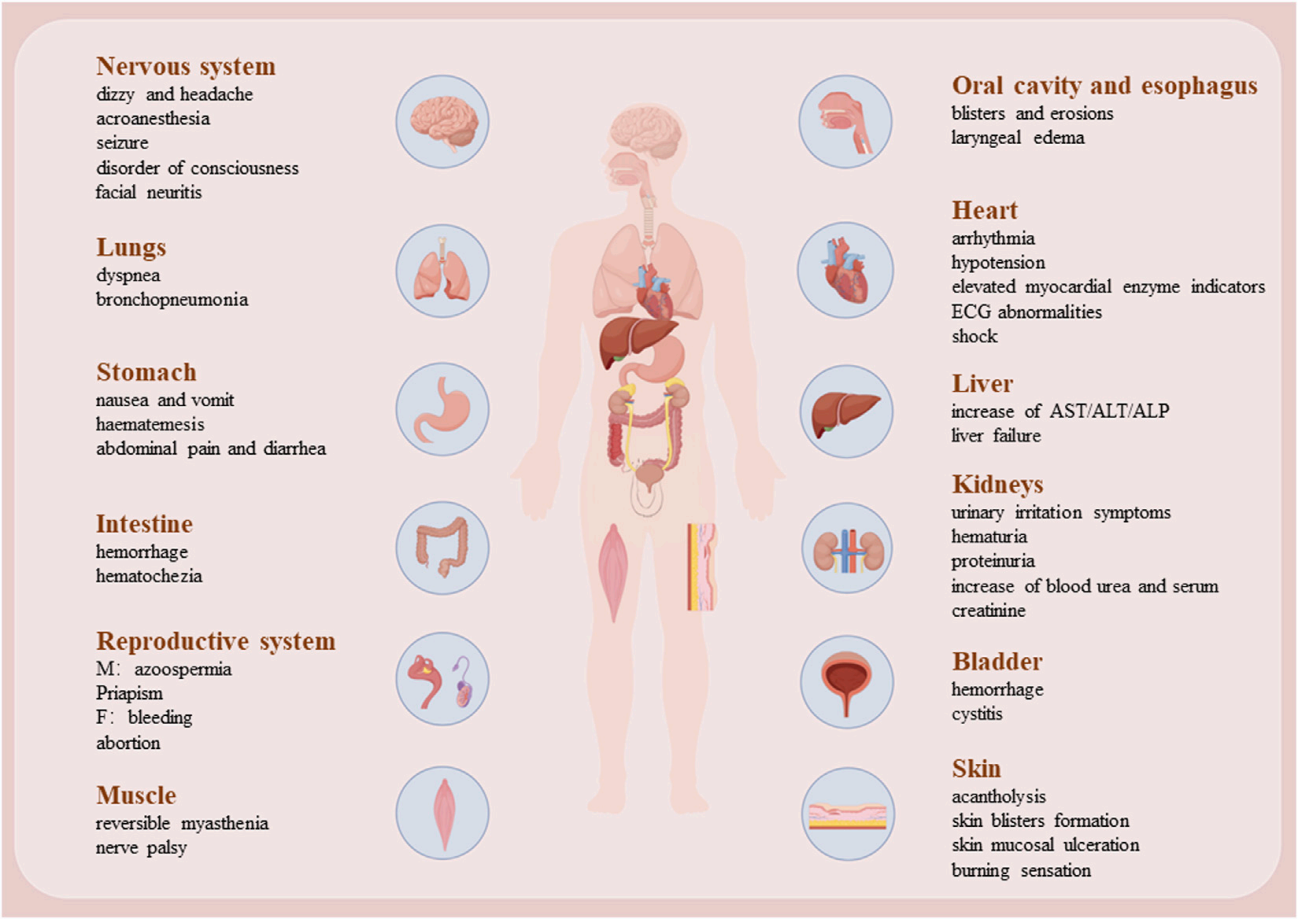
The damage caused by CTD to various organs.

Although cantharidin exhibits significant pharmacological effects, its toxic side effects are substantial and warrant attention. Our research group previously compiled the adverse toxicity events associated with cantharidin, revealing that it exerts toxic effects on various bodily organs, especially the heart, liver, and kidneys (Zhang Y. et al., 2018). Previous experimental studies by our group have demonstrated that cantharidin induces acute cardiac and hepatic toxicity (Youyou et al., 2020; Zhang et al., 2020; Yu et al., 2020). This toxicity primarily arises from its ability to trigger necrosis and apoptosis in cardiomyocytes and hepatocytes, while also promoting inflammation. Associated clinical symptoms include shock, gastrointestinal and urinary tract irritation, renal insufficiency, hematuria, heart failure, and hypocalcemia. These toxicity manifestations are summarized in Figure 4.

### 3.1 Cardiotoxicity

In case of large-dose CTD poisoning, myocardial injury manifests earlier than hepatic and kidney functional abnormalities. Excessive exposure can lead to direct cardiotoxic effects, including arrhythmias and arterial occlusion (Rabkin et al., 1979). Severe poisoning elevates cardiac enzyme levels, notably serum creatine kinase (Zhang Y. et al., 2018; Youyou et al., 2020). Previous studies on fatal CTD poisoning have shown ventricular arrhythmias, persistent ST-segment elevation, and T-wave alterations on electrocardiography, accompanied by anatomical evidence of myocardial hemorrhage and patchy myocardial necrosis. These ST-segment and T-wave alterations correlate with focal myocardial degeneration, necrosis, inflammation, and/or fibrosis. However, as with other cardiomyopathies, the precise mechanisms of CTD’s cardiotoxicity remain elusive. Research by multiple author (Rabkin et al., 1979; Shao et al., 2020) has identified dose-dependent mitochondrial swelling, nuclear chromatin clumping, sarcoplasmic reticulum disruption, and myofibrillar degeneration in CTD-poisoned myocardium. These electron microscopic observations suggest severe mitochondrial damage as a key feature of CTD-induced myocardial injury. Fleckenstein et al. (1975) proposed that myocardial necrosis results from excessive calcium influx into myocardial cells, with electron-dense structures observed within mitochondria potentially representing calcium deposits. This offers a plausible explanation for CTD-induced myocardial injury, where CTD alters myocardial cell membranes or mitochondria, leading to calcium overload and ultimately myocardial fiber necrosis. Shi et al. reported that CTD impairs mitochondrial function, evidenced by reduced basal respiration, ATP levels, and spare respiratory capacity, alongside decreased mitochondrial DNA copy numbers and downregulated mRNA levels of cytochrome C oxidase I, II, and III. Additionally, CTD inhibited activities of mitochondrial complexes I and II (Shi et al., 2022). Youyou et al. (2020) previously validated factors related to hypoxia, angiogenesis, and apoptosis, which confirmed that CTD can induce hypoxia, necrosis, and inflammation. Shao et al. (2020) observed early oxidative stress parameters affecting myocardium in CTD poisoning, marked by significant increases in superoxide dismutase and glutathione levels, coupled with a notable decrease in malondialdehyde content. Transcriptomic analysis underscored the roles of *Tnc* and *Myh7* genes in CTD-induced myocardial injury in rats, involving vascular regeneration, myocardial repair, and remodeling (Youyou et al., 2020). Additionally, CTD poisoning may directly impair capillary endothelial cells, leading to widened intercellular spaces and increased vascular permeability. This facilitates the extravasation of plasma components, contributing to myocardial cells and myocardial hemorrhage (Knapp et al., 1999). In conclusion, the mitochondrial damage and cardiomyocyte apoptosis induced by CTD are significant and warrant attention. As a phosphatase inhibitor, CTD disrupts multiple energy pathways in cardiomyocytes, potentially leading to arrhythmia and, in severe cases, sudden death. Shao et al. (2020) provided evidence that L-glutamine could protect cardiac cells against the acute cantharidin-induced cardiotoxicity, and the protective mechanism of glutamine may be related to the myocardial cell membrane or the tricarboxylic acid cycle in the mitochondria.

### 3.2 Hepatotoxicity

The liver, as the primary organ responsible for drug metabolism and detoxification, is more susceptible to drug-induced damage compared to other organs. Zhang Y. et al. (2018) reported varying degrees of liver injury in retrospective study of CTD poisoning and associated mortality cases. Serum biochemical markers of liver injury, such as bilirubin, aspartate aminotransferase (AST), alanine aminotransferase (ALT), and alkaline phosphatase (ALP), were significantly elevated in models of different degrees of CTD-induced liver injury and toxicity. Post-mortem examinations of cadavers revealed hepatic infiltration by inflammatory cells, hepatocyte degeneration, and necrosis. In rat models, high doses of CTD triggered multifocal hepatic hemorrhage, focal necrosis, and inflammatory cell infiltration (Yu et al., 2020). These observations were substantiated by Western blot analysis, which revealed increased inflammatory factors and confirmed hepatocyte apoptosis and necrosis following CTD exposure. Jin et al. (2023) outlined that CTD-mediated hepatotoxicity mainly involves endoplasmic reticulum stress (ERS), autophagy, activation of cysteine signaling pathways, mitochondrial dysfunction, and bile acid circulation. CTD induces ERS, mitochondrial swelling and distortion, altering hepatocyte membrane permeability and ultimately cell death, thereby causing liver damage. Metabolomic analysis via UPLC-Q-TOF/MS (Zhu et al., 2019) indicated that CTD treatment disrupts 14 metabolic pathways, indicating its potential to impair various biological processes within the liver. Comparative studies further identified significant perturbations in glutathione metabolism, taurine and hypotaurine metabolism, and the interconversion of pentose and glucuronate. Additionally, metabolic pathway analysis identified CTD-induced disturbances in serum lipid metabolism, pointing to dysregulation in biosynthetic pathways anchored by glycerophospholipids, glycerides, and glycosylphosphatidylinositol (GPI) (Li et al., 2024). Cytochrome P450 enzymes play a crucial role in hepatic drug metabolism, with CTD shown to inhibits CYP2D6 and CYP3A4 while inducing CYP2C9 (Zhou et al., 2015). Consequently, caution is warranted when co-administering CTD with drugs primarily reliant on the cytochrome P450 enzyme system for metabolism. Interestingly, vitamin C (VC) has been found to attenuate liver damage by downregulating CTD-induced mRNA expression of TLR4 and NF-κB in the liver, participating in the inhibition of intrahepatic oxidative stress and the TLR4/NF-κB pathway associated with inflammation, thereby improving hepatocyte metabolic function (Wu et al., 2015). Ginsenoside Rb1 (GRb1) shows potential hepatoprotective effects. Xiong et al. revealed the mechanism of GRb1 against CTD-induced hepatotoxicity by inhibiting apoptosis and endoplasmic reticulum stress (ERS) (Xiong et al., 2024). Similarly, Astragalus polysaccharides (APS) regulated primary bile acid biosynthesis and glycerophospholipid metabolism, thus decreasing the liver damage caused by CTD (Huang et al., 2021). GRb1 and APS are present in many clinically used drugs to reduce the hepatotoxicity caused by CTD (Jin et al., 2023).

### 3.3 Renal toxicity

CTD poisoning frequently manifests as lumbago and renal dysfunction, evidenced by elevated serum creatinine and urea levels. AS the kidneys metabolized CTD, it may irritate the urinary tract, leading to conditions such as proteinuria, glucosuria, and hematuria (Cotovio et al., 2013). Anatomically, affected kidneys often appear pale, with histological changes including renal capsule hemorrhage, interstitial bleeding, and basal membrane edema (Karras et al., 1996). Common histopathological features include acute tubular necrosis, epithelial cell shedding, and tubular casts. In murine models, CTD exposure induces vacuolar degeneration of renal tubular epithelial cells, glomerular swelling, and interstitial inflammatory cell infiltration. Ultrastructural studies further reveal mitochondrial swelling, disrupted mitochondrial cristae, and increased autophagic vacuoles. The main mechanism likely involves CTD binding to albumin, leading to glomerular injury and acute tubular necrosis upon filtration through the renal glomeruli. CTD triggers endoplasmic reticulum stress (ERS), potentially driving renal toxicity via activation of autophagy and cell apoptosis, mediated through the PERK/CHOP pathway dependent on ERS (He et al., 2022). Transcriptomic studies reveal that CTD alters gene expression related to stress response, cell death induction, transporter proteins, and affects signaling pathways like MAPK, AMPK, and HIF-1 (Liu et al., 2023). These changes contribute to renal oxidative stress, inflammation, autophagy, and apoptosis. Using network pharmacology and UHPLC-QE/MS-based metabolomics, Tianmu He et al. demonstrated that CTD increases Caspase 3 expression levels and the Bax/Bcl-2 ratio in renal cells. Additionally, CTD appears to inhibit glycerophospholipid and sphingolipid pathways, phosphatidylethanolamine, phosphatidylcholine, MAPK3, and PLD2 (He et al., 2020). The recent study (He et al., 2025) showed that CTD induces acute tubular necrosis (ATN), resulting in acute kidney injury (AKI), by activating glycerophospholipid (GP) metabolism and inhibiting sphingolipid (SL)_metabolism in the renal cortex and medulla. In animal models, Ginsenoside Rb1 markedly alleviated cantharidin-induced acute kidney injury by modulating toll-like receptor 4 dimerization and NF-kB/MAPKs signaling pathways (Gao et al., 2020).

### 3.4 Reproductive toxicity

While the deleterious effects of CTD on cardiac, hepatic, and renal functions have been well-documented, its reproductive toxicity remains understudied. Historically, CTD was employed in Europe as an aphrodisiac due to its effects on penile congestion and erection in males, and vaginal bleeding in females (Karras et al., 1996). In murine models, CTD exposure drastically reduces testicular index and serum testosterone levels (Xiao et al., 2024). Histological examination reveals a significant decrease in sperm count within seminiferous tubules, accompanied by vacuolization, necrosis of spermatogenic cells, and peripheral infiltration of inflammatory cells. Testicular tissues also exhibit notable oxidative damage and increased mitochondrial autophagy, with suppression of the Nrf2-Keap1 pathway and disruption of the blood-testis barrier. Liu et al. reported that exposure to varying concentrations of CTD induces weight loss and increases testicular coefficient (Liu et al., 2024). Network toxicology and Western blot analyses indicate inhibition of PI3K, AKT, and anti-apoptotic protein Bcl-2 expression, alongside promotion of pro-apoptotic proteins Bax and Caspase-3. Pathway enrichment analysis highlights associations between CTD-induced testicular damage, apoptosis, and the PI3K/AKT and HIF-1 signaling pathways. Regarding CTD’s potential role in abortion, Nae-Fang Twu et al. explored its impact on steroidogenesis in goat corpus luteum cells, revealing a reduction in steroids and progesterone (P4) production, underscoring CTD’s potential reproductive toxicity (Twu et al., 2012). Study had shown that Astragalus polysaccharide can attenuate cantharidin-induced toxic damage, oxidative stress and autophagy in the testes of mice. These protective effects may be closely mediated by regulating the Nrf2-Keap1 signaling pathway, inhibiting autophagy, and restoring the blood-testis barrier (Xiao et al., 2024).

### 3.5 Other toxicities

CTD exhibits significant irritant effects on skin and mucous membranes, leading to desquamation (Karras et al., 1996), epidermal vesicles, submucosal edema, hemorrhage, necrosis, and sloughing. Ingestion of beetles containing CTD can cause oral burning sensation, dry retching, vomiting, and diffuse abdominal pain (Cotovio et al., 2013), with potential progression to acute gastrointestinal inflammation or shock. Histopathological findings include esophageal mucosal congestion, swelling, and ulcers, as well as gastric mucosal hyperemia, hemorrhage, and superficial erosions extending to the upper small intestine (Chen and Huang). CTD residues are often detectable in the stomach, which aids in forensic identification of CTD poisoning. Neurological effects resembling Guillain-Barré syndrome have been reported following CTD exposure, suggesting potential neurotoxicity (Harrisberg et al., 1984). Additionally, reversible impairments like cranial nerve paralysis and muscle weakness have been documented, with neuronal recovery typically occurring after treatment (Zouvanis et al., 1994). In studies using hippocampal brain slices, CTD exposure was found to reduce basal synaptic transmission, disrupts synaptic plasticity induction via inconsistent effects on Ca^2+^ signaling across different concentrations, and indicates long-term synaptic transmission reduction through prolonged protein phosphatase inhibition (Koss et al., 2007). CTD also affects the immune system by impairing differentiation and maturation phenotypes of dendritic cells (DCs), as evidenced by downregulation of surface markers CD1a, CD83, and DC-SIGN. Cases of CTD-induced cystitis have been observed (Chen and Teik, 1961), and animal studies have demonstrated severe inflammatory bladder injury with hematuria, possibly mediated by c-Fos and COX-2 overexpression (Huan et al., 2012). In conclusion, while CTD offers considerable therapeutic potential for dermatological and oncological applications, its clinical use demands stringent control over dosage and treatment duration to minimize its toxic side effects.

### 3.6 Animal toxicities

The toxic effects of CTD in animals are similar to those in humans. Toxicosis occurs when horses consume alfalfa hay or products contaminated with “blister” beetles (Schmitz, 1989). In horses that remain alive for several days, persistence of elevated heart and respiratory rates and increasing serum creatine kinase concentration are associated with a deteriorating condition. Treatment of CTD toxicosis is symptomatic, with gastrointestinal protectants, laxative, intravenous fluids, analgesics, diuretics, calcium gluconate, and magnesium are all included in the treatment regimen. In experiments investigating (Qualls et al., 2013) the use of mineral oil, charcoal, and montmorillonite for treating oral CTD poisoning, it was found that mineral oil exacerbates the toxic effects of CTD. This may be attributed to the fact that CTD is fat-soluble; dissolving it in mineral oil enhances its absorption. This finding also indicates that mineral oil should not be used in the treatment of oral CTD poisoning in animals.

Currently, there is no specific antidote for CTD poisoning. The treatment after CTD poisoning mainly focuses on protecting the gastrointestinal mucosa, performing blood purification, and safeguarding the functions of the heart, lungs, kidneys, and brain. For those who have ingested CTD orally, gastric lavage should be carried out as early as possible. Patients at risk of acute kidney injury should have their renal function closely monitored, and protective measures such as urine alkalization, the use of diuretics (e.g., furosemide), and avoiding the use of nephrotoxic drugs should be taken (Duan et al., 2025). Given that hemodialysis is ineffective in removing CTD, owing to its binding to circulating albumin and poor aqueous solubility, hemoperfusion and hemofiltration are recommended as alternative approaches. These methods can facilitate the removal of CTD, inflammatory cytokines, and metabolites, while also correcting electrolyte and acid-base disturbances (Xu et al., 2024). In addition, cardiac and liver function indicators should be observed, and targeted treatment should be provided. Although CTD is metabolized relatively quickly in the early stage, the original substance can still be detected in the body after a few days. Therefore, sequelae should also be monitored, and gastroenteritis should be noted in patients who have ingested it orally. In summary, CTD poisoning warrants serious attention. It is advisable to not only administer targeted symptomatic treatment but also closely monitor potential systemic harm to the human body.

## 4 Chemical analysis and toxicological data of CTD

Following oral administration, CTD is rapidly absorbed into the bloodstream and extensively distributed into tissues, facilitated by its lipophilicity and strong penetrability. Topical applcation also results in rapid systemic absorption, as evidenced by documented fatalities from external application reported by Chinese scholars. In beagle dogs, intravenous administration of CTD results in swift distribution across various organ tissues and rapid elimination, with a half-life of 0.69 ± 0.03 h (Dang and Zhu, 2009). Pharmacokinetic study of CTD in rats demonstrated rapid plasma clearance of CTD, with higher concentrations in the liver and kidney compared to cardiac tissues (Duan et al., 2021). Although CTD concentrations in the heart and spleen are lower than in the liver and kidneys, these organs exhibit slower metabolism, potentially due to insufficient metabolic or detoxifying enzyme activity. In case of fatal poisoning, blood is usually extracted for quantitative detection of toxins. Urine, however, serves as another valuable specimen for routine post-poisoning analysis, although CTD concentrations decline significantly 3–4 days after ingestion. Therefore, early urine collection in the course of the illness is crucial for diagnosis accuracy (Xiao et al., 2019). Analysis of blood and urine from a CTD poisoning victim prior to death detected unmodified compounds 30 h post-ingestion (Polettini et al., 1992), indicating that CTD undergoes rapid early metabolism, followed by a slower metabolism in the later stages. In forensic cases of CTD poisoning, *postmortem* blood is typically sampled for toxicological analysis. However, given CTD’s short half-life, liver and kidney tissues are preferred for quantitative analysis, with heart and spleen tissues also serving as suitable detection targets.

It has been reported that most *Cantharis* species contain approximately 1%–1.2% (0.2–0.7 mg) of CTD (Al-Binali et al., 2010). When ingested orally, 0.6–1 g of blister beetles is typically toxic, while 1.5–3 g (from about 5 individuals) can be lethal (Tagwireyi et al., 2000). The therapeutic dose of CTD is very close to its toxic dose, with fatal oral doses ranging from 10–60 mg, and a median lethal dose (LD50) in mice of 1.71 mg/kg (Jin et al., 2023). Polettini (Polettini et al., 1992) utilized GC-MS to analyze that approximately 1.3% of the active ingredients in the powder were CTD, estimating a fatal intake of 26–45 mg of CTD. Other scholars (Hundt et al., 1990), using GC/MS analysis, reported *postmortem* serum contains of 0.0723 ug/mL in cases of CTD poisoning, with CTD powder containing 0.87% CTD. In cases reported by Cheng, where CTD was used for abortion, blood concentrations of CTD were 0.27 ug/mL antemortem and 0.11 ug/mL *postmortem* (Cheng et al., 1990). Zhang (Zhang Y. et al., 2018) reviewed cases of CTD poisoning in China, reporting an average toxic dose of CTD powder of 3.36 ± 4.01 g, an average intoxication time of 628.44 ± 1166.60 min, and 17 fatalities with an average toxic dose of 8.79 ± 5.42 g. Most deaths occurred within 24 h, with poisoning-induced renal failure and acute circulatory failure identified as the primary causes. However, this study did not include qualitative and quantitative analyses of *postmortem* serum or organs. We have compiled reports of CTD poisoning by Chinese scholars and summarized clinical manifestations and related data in Table 1. Among these cases, only one included quantitative testing. Since CTD powder is derived from ground *Cantharis* individuals, combining the findings of the aforementioned studies suggests that the toxic dose of CTD is approximately 29.2 ± 34.8 mg, with an estimated fatal dose of 76.4 ± 47.1 mg. Variations in reported blood concentrations may be attributed to differences in the time elapsed since death and the timing of *postmortem* testing. In China, numerous cases of successful recovery from CTD poisoning have been documented, potentially reflecting individual differences in CTD metabolism. Clinicians should be aware of this variability in clinical practice. Subsequent studies could explore the impact of detoxification enzymes, analogous to the relationship between aldehyde dehydrogenase and alcohol metabolism.

**TABLE 1:** Basic information of reported cases of CTD poison in China.

No.	Age/Sex	Primary diseases	Dosage and medication method	Survival time	Toxicological analysis	Clinical and autopsy information	
						Clinical information	Autopsy information
1 (Huang and Xian, 1995)	44/F	Fatigue	6g/oral	3 days	-	Dizziness, perioral numbness, nausea, muscle rigidity, twitching of the extremities, myocardial impairment, Mobitz type II AV block, acute kidney failure	no autopsy
2 (Tian et al., 1982)	48/F	Stomach discomfort	1.66 g for 3days/oral	4 days	Qualitative	Blister formation on the inner thighs and lateral lower leg, water and electrolyte disturbances, oliguria, acute kidney failure	Pulmonary edema and myocardial necrosis, scattered bleeding points on the kidney capsule surface, glomerulosclerosis, renal tubular type, renal interstitial hemorrhage
3 (Chi and Ma, 1964)	28/F	Mistake	3g/oral	34 h	-	Nausea and vomiting, abdominal pain, urination with burning, urinary frequency and oliguria, low blood pressure, urine protein, normal liver function	no autopsy
4 (Zou, 1999)	21/F	Abortion	25g/oral	24 h	Qualitative	Nausea and vomiting, P 140/min, low blood pressure, generalized cutaneous blisters	gastric and small intestinal mucosal necrosis, gastric mucosal bleeding, myocardial fiber rupture, necrosis of hepatocytes, mild multifocal acute necrosis of tubular epithelial cells in kidneys
5 (Tan, 2002)	43/F	Skin ulcers	-/oral	-	Qualitative	Nausea and vomiting, abdominal pain, urinary frequency and oliguria	gastric and small intestinal mucosal hemorrhage, swollen renal epithelial tubular cells, fibrin exudation in the lungs
6 (Zhang et al., 2012)	10/F	Psoriasis	-/extern	25 h	0.1 ug/mL in stomach by GC-MS (may be caused by *postmortem* diffusion)	Headache, nausea and vomiting, blister formation	no autopsy
7 (Li et al., 2008)	34/F	Psoriasis	-/extern	5 d	Qualitative	Vomiting, abdominal pain, urinary frequency and oliguria, urination with burning, skin ulcer	Neural cell death and calcification, focal cardiac myocyte dissolution, kidney swelling, necrosis of renal tubular epithelial cells and glomerular atrophy, renal tubular type, hemorrhage of the bladder mucosa
8 (Shi and Wang, 2003)	39/M	Psoriasis	20g/extern (25% of body surface area)	22 h	90.7 μg/m、130 μg/g、53 μg/g and 1.8 mg/g in the blood, skin, kidney and medicine	Blister formation, vomiting	Scattered bleeding points in the aortic root and surface of heart, necrosis of renal tubular epithelial cells, renal tubular type, small intestinal mucosal erosion, hepatic bridging necrosis with infiltration of neutrophils, plasma cells, lymphocytes
9 (Yang and Wang, 2005)	28/F	lymphadenopathy	3g/oral/3 d	3 days	Qualitative (GC/MS)	Headache, nausea and vomiting, abdominal pain, hematuresis	Cardiomyocyte hypertrophy, esophageal and gastric mucosal bleeding, hemorrhage of the bladder mucosa, necrosis of renal tubular epithelial cells and glomerular atrophy, renal tubular type
10 (Sun and Shi, 2009)	24/F	Psoriasis	10g/extern	2 days	Qualitative	Nausea and vomiting, blister formation	Hemorrhages in subepicardial and subserosal areas, pneumonia, hydropic degeneration of hepatocytes, necrosis of renal tubular epithelial cells, renal tubular type, microthrombi formation within glomerular capillaries
11 (Peng and Liang, 2015)	56/M	Dog bite	5g/oral	2 days	Qualitative	Nausea and vomiting, abdominal pain, hematuresis, hematochezia	Hemorrhages in subepicardial and subserosal areas, scattered bleeding points in the surface of heart
12 (Li and Dai, 1986)	8/M	Dog bite	6g/oral	24 h	-	Nausea and vomiting, P 160/min, bloody nasal discharge	Scattered bleeding points in the surface of left ventricle, myocardial interstitial hemorrhage, some broken and dissolved myocardial fibers, necrosis of renal tubular epithelial cells, renal tubular type, renal interstitial hemorrhage
13 (Xun, 1990)	21/22/F	Abortion	30 g/oral 3 g/oral and 2 g/extern	4h/9 h	Qualitative	Ulceration in the oral mucosa, blister formation, nausea and vomiting, abdominal pain, bloody vaginal discharge	Esophageal and gastric mucosal bleeding
14 (Chen, 1981)	34/24/23/20/32/F	-	-	24h/2h/2h/2h/3 days	-	Haemorrhage (1), bloody vaginal discharge (1), nausea and vomiting (3), blister formation (5)	bloated cadaver (3)

## 5 Modification of the structure of CTD

**FIGURE 5:**
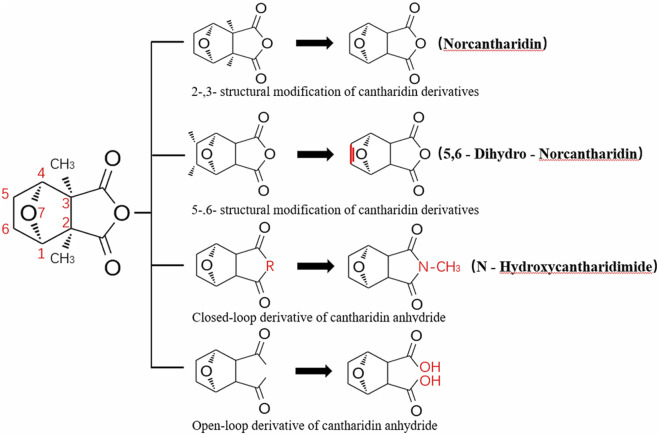
Structural modification of cantharidin derivatives.

CTD exhibits strong toxicity to mammals, which limits its clinical application. Additionally, it has poor solubility and a low gastrointestinal bioavailability of 26.7% (Puerto Galvis et al., 2013). Consequently, achieving effective results necessitates high dosages, which in turn result in significant toxicity. Therefore, current research efforts are focused on reducing its toxicity. CTD consists of a six-ring and a five-ring, the oxygen parts on the six-ring and anhydride section exhibit biochemical activity. Although the mechanism of toxicity of CTD is not fully understood, structural modifications of CTD have led to the development of new drugs for clinical use. Many analogs, such as (NCTD) and CTD amide compounds (Wang et al., 2018), have been synthetically produced, demonstrating improved therapeutic effects by reducing inherent toxicity while retaining biological activity, particularly in anti-cancer applications. We will conduct a review on different connection sites of the six-membered ring in CTD and the structural modification of the five-membered ring, aiming to provide references for subsequent research (Figure 5).

### 5.1 Modification on C-1,4 and 7-O position

Through the study on the structure-activity relationship of modifications at positions 1 and 4 of the CTD structure (Figure 5; Table 2) (McCluskey et al., 2003; Ren and Kinghorn, 2021; McCuskey et al., 2000), it was found that the structural modifications have a significant site-dependence on the phosphatase inhibitory activity. Experimental data indicate that substitution modification at position 1 reduces the inhibitory activity of the derivatives against PP2A, whereas single substitution at position 4 diminishes their broad-spectrum inhibitory capacity against protein phosphatases. Mccluskey et al. (1996) has synthesized a series of CTD analogs, indicating that the 7-hydroxy group is essential for inhibiting PP2A. The derivatives obtained by replacing oxygen atoms with sulfur atoms also lose their inhibitory effect on PPs (Shan et al., 2006). Based on this, these core pharmacophore groups at positions C-1,4 and 7-O should be preferentially retained during the structural optimization of CTD.

**TABLE 2:** Common methods for structural modification of CTD.

Modification positions	Compounds	Name	IC_50_ (μmol/L)	Cells	Inhibition of PPs (IC_50_,μmol/l)		PP2A inhibition rate (%)	Pharmacology (compered with CTD)	Toxicity (compered with CTD)	LD_50_ (mouse, mg/kg)	References
					PP1	PP2A					
-	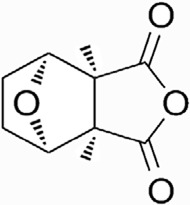	CTD	19.1	HepG2	0.47	0.04	92–95	-	-	1.0	Ren and Kinghorn (2021), McCuskey et al. (2000)
Modification on C-1,4 Positions	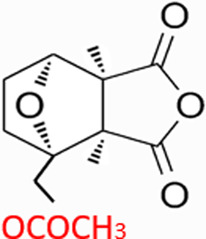	-	≥1000	HepG2	≥1000	≥1000	-	Decrease	-	-	Shan et al. (2006), Baba et al. (2003)
	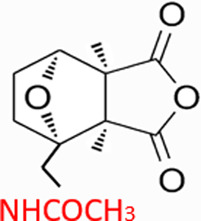	-	≥1000	HepG2	≥1000	≥1000	-	Decrease	-	-	Shan et al. (2006)
Modification on C-2,3 Positions	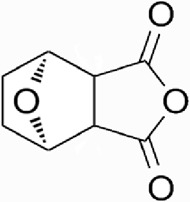	Norcantharidin	212.9 ± 26.2/2.71	HepG2/L1210	1.98	0.37	-	Increase	Decrease	4.0	Ren and Kinghorn (2021), Shan et al. (2006)
	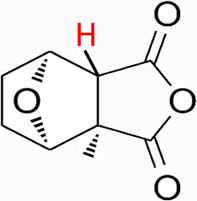	(*S*)-palasonin	-	-	0.66	0.12	-	Similar	Decrease	-	Ren and Kinghorn (2021)
	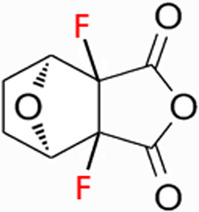	2,3-Difluoronorcantharidin	6.19	L1210	-	-	-	Similar	Decrease	-	Essers et al. (2001)
Modification on C-5,6 Positions	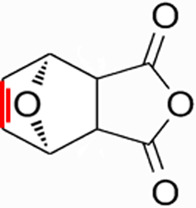	Unsaturated Norcantharidin	245	HepG2	-	-	97	Similar	Decrease	-	McCuskey et al. (2000), McCluskey et al. (2000)
	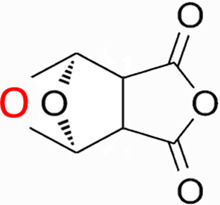	Δ-5,6-ethyl norcantharidin	≥1000	-	≥1000	≥1000	-	Decrease	-	-	Thaqi et al. (2010)
Modification on C-7 Positions	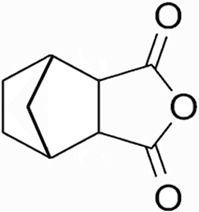	Hexahydro-4,7-methanoisobenzofuran-1,3-dione	-	-	≥1000	≥1000	38.7	Decrease	-	-	Mccluskey et al. (1996)
Analogues that Maintain the Five-Membered ring	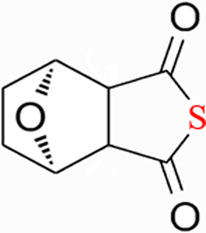	2,3-dimethyl endothall thioanhydrid	14.7 ± 7.0	HepG2	3.05± 1.05	2.11± 0.26	-	Similar	Similar	-	Shan et al. (2006)
	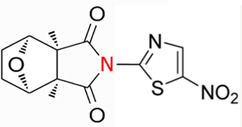	*N*-[2-(5-nitrothiazolyl)]cantharidinimide	0.4	Hep3B	-	-	-	Increase	Decrease	-	Ren and Kinghorn (2021)
Analogues that Open the Five-Membered Ring	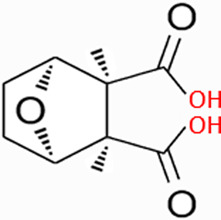	Cantharidic acid	218.5		0.56	0.05	92–95	Similar	Decrease	1.8	Ren and Kinghorn (2021), McCuskey et al. (2000)
	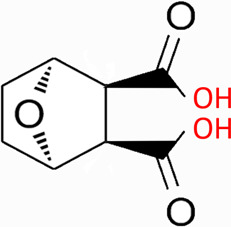	Endothall	-	-	5.0	0.97	-	Similar	Decrease	14	Ren and Kinghorn (2021)
	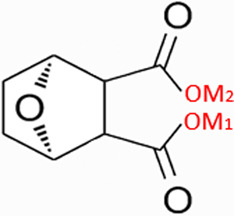	Metal Salt Analogues a.M1 = Na,M2 = K b.M1 = Mg,M2 = K c.M1 = Mg,M2 = Na d.M1 = Ba,M2 = K e.M1 = Ba,M2 = Na	a = 67.37% b = 70.77% c = 68.25% d = 71.79% e = 70.41%	HepG2	-	-	-	Similar	Decreased Toxicity, and increased water solubility	-	Zhao et al. (2019)

### 5.2 Modification on C-2,3 position

Norcantharidin (NCTD) is a synthetic anti-cancer compound derived from CTD. It is produced by removing methyl groups from C-2 and C-3 positions of CTD through hydrolysis, which reduces nephrotoxicity while maintaining anti-cancer activity comparable to that of CTD (Hizartz et al., 2019; Massicot et al., 2005). Compared to CTD, NCTD exhibits lower cytotoxicity and fewer clinical side effects. Since 1984, NCTD has been utilized in China for the treatment of various cancers. It shows stronger inhibition against PP2B compared to CTD, demonstrating its potent activity against tumor cells. NCTD is particularly effective in treating primary liver cancer, where it enhances immune function and reduces tumor size in early to mid-stage cases (Li et al., 2010; Zhao et al., 2019). Research involving BEL-7402 liver cells has shown that NCTD inhibit NF-κB activation, a pathway that protects tumor cells from apoptosis (Zavoral et al., 2011). To further mitigate its toxicity, ongoing studies are exploring the use of thermosensitive hydrogel nanoparticles to encapsulate NCTD, aiming to enhance its efficacy while reducing required dosage (Li XY. et al., 2021).

Essers introduced fluorine groups at the C-2,3 positions, and the derivatives retained the antitumor activity and simultaneously reduced the toxic effects (Essers et al., 2001). In addition, the hydrogen atoms or other groups can also be used for substitution, and the synthesized derivatives show an increased affinity for PPs (Laidley et al., 1999). Furthermore, modifications can tailor the affinity of CTD analogs for different subtypes of serine/threonine phosphatases, enabling targeted enzyme inhibition. Yoshiyasu Baba developed a novel derivative, 1,5-diphenylacetyl oxyethyl substituted NCTD, which selectively inhibits PP2B without affecting PP1 or PP2A (Baba et al., 2003).

### 5.3 Modification on C-5,6 position

Dehydrogenation at the fifth and sixth positions of CTD forms a C=C double bond, yielding a derivative (McCluskey et al., 2000) that exhibits anti-cancer activity comparable to CTD but with reduced toxicity. This derivative enables the introduction of hydrophobic groups such as methyl or ester groups, or epoxy structures. Experimental studies by Thaqi have shown that related analogs lacking the 5,6-ethylene bridge display poorer inhibitory activity, confirming the crucial role of this bridge in biological activity (Thaqi et al., 2010).

### 5.4 Acid Anhydride-Modified derivatives

Anhydride-Modified derivatives of CTD through anhydride modifications yield various analogues (Wang Y. et al., 2015). These derivatives, modified in the anhydride moiety, can be categorized into two categories: those that retain the five-membered anhydride ring and those that involve five-membered ring opening.

McCluskey demonstrated through research that introducing a nitrogen atom into the five-membered ring can lead to the loss of the effect on PP2A. However, introducing a sulfur atom results in an inhibitory effect on PP2A similar to that of CTD and NCTD (McCuskey et al., 2000). Research has shown that maintaining the five-membered ring while substituting with specific groups can produce compounds like cantharimides, a novel class of CTD analogues exhibiting superior inhibition of PP1 and PP2A compared to NCTD (McCluskey et al., 2001). These cantharimides also possess improved solubility and feature electron-withdrawing groups on the aromatic ring, which enhance cytotoxicity against cancer cell lines.

Regarding the analogs with an opened five - membered ring, metal salt analogs are currently a hot research topic. NCT sodium salt is a newly developed drug, and the sodium atom can be substituted with various metal atoms. These NCT metal salt analogs have a strong inhibitory effect on tumors and hold broad application prospects (Zhao et al., 2019). Additionally, a series of ring-opened CTD analogues with only one free carboxylate has been developed, retaining inhibitory activity against PP1 and PP2A while slightly increasing selectivity towards PP2A (McCluskey et al., 2001). The above two derivatives with an opened five - membered ring are mainly modified to address the weaknesses of cantharidin, such as its poor bioavailability and water solubility (Zhao et al., 2019). The water solubility of the derivatives is increased, their inhibitory effect on tumors is also enhanced, and their toxic effects are reduced. Notably, studies by Ji-Yeon Lee explored N-Benzylcantharidinamide, demonstrating its ability to inhibit MMP-9 expression by reducing MMP-9 mRNA stability without affecting MMP-9 transcriptional activity (Lee et al., 2014). This finding suggests potential for preventing tumor invasion and metastasis. Modifications to the anhydride five-membered ring do not universally enhance PP2A inhibition, in some cases, they may even negate the original activity (McCuskey et al., 2000). Conversely, research by Adam McCluskey has shown that derivatives capable of facile ring opening of the anhydride moiety retain significant PP2A inhibition and anti-cancer activity (McCuskey et al., 2000). However, the degree of inhibition on PP2A has decreased.

In addition, researches had also been conducted on the carriers of CTD and its derivatives. Drug-loaded liposomes have attracted much attention due to their advantages such as enhancing activity, improving bioavailability, having low toxicity, and enhancing targeting (Wang et al., 2021). Multiple studies have been carried out to explore the efficacy of carriers for CTD, NCTD and their analogs, such as liposomes, nanoparticles, and micelles (Zhu et al., 2018; Zhou et al., 2019; Zhang H. et al., 2018; Yao et al., 2020). These carriers enhance targeting and reduce toxic side effects. Moreover, as mentioned earlier, when some other substances are used in combination with CTD, such as APS and L-glutamine (Shao et al., 2020; Huang et al., 2021), they can reduce the liver and heart toxicity caused by CTD.

In summary, current modifications of CTD primarily focus on its six-membered and five-membered rings, with the first, fourth, and seventh hydroxy groups on the six-membered ring being crucial factors, modifications at these positions should be avoided in the future. Removing the methyl groups at the C-2 and C-3 positions of cantharidin or substituting them with other groups can significantly reduce the toxic effects and result in better selectivity. Similarly, the C-5, 6 ethyl bridge position is crucial. Forming a C=C double bond or introducing hydrophobic groups at this position can reduce toxic side effects and enhance the inhibitory effect on tumor cells. For the five-membered ring, introducing a carboxylic acid group or a metal salt can increase the water solubility of CTD and its derivatives, enhance absorption, and reduce toxicity. Moreover, after opening the five-membered ring, the inhibitory effect on PP2A is lower compared to the closed-ring form, which is a point worthy of attention. Moreover, “packaging” strategies for CTD, such as liposome encapsulation, have shown promising anti-proliferative effects against cancer cells.

## 6 Conclusion

In recent years, with deepening research into CTD, researchers have discovered its broad pharmacological effects and significant clinical potential, particularly in oncology, where it demonstrates extensive anti-tumor activity and promising applications. The core mechanism lies in its ability to inhibit the activity of protein phosphatases, thereby causing changes in the internal mechanisms of tumor cells. While it brings benefits, it also damages the functions of organs in the human body.

Here, we provide a comprehensive review of CTD, covering its pharmacology, toxicology, toxic dose, and derivatives. In particular, it summarizes cantharidin’s cardiovascular effects, its role in the agricultural field, as well as the symptoms, mechanisms, and toxic dose of CTD poisoning. Additionally, we analyze the structure of CTD, offering reference suggestions for future modifications of cantharidin. However, due to space constraints, this thesis cannot list all mechanisms and only summarizes the important ones. Future studies can focus on a specific property of cantharidin for a dedicated summary.

In the future, we should focus on the modification of CTD, retaining the active groups and removing the toxic groups in its structure. With the development of new materials such as nanomaterials, we can develop advanced drug carriers and targeted drug-delivery systems, and explore bacterial biotransformation. Moreover, for the use of CTD and its derivatives, standardized management should be carried out, and corresponding clinical use manuals should be compiled. While strictly restricting its clinical use, treatment for its toxic effects should be well-prepared. Research on the combined therapy involving CTD and other drugs also has great prospects, which is expected to provide theoretical basis and practical guidance for the development of safer and more effective therapeutic drugs.

## Glossary

ADP: Adenosine diphosphate
ALP: Alkaline phosphatase
ALT: Alanine aminotransferase
AST: Aspartate aminotransferase
ATP: Adenosine triphosphate
Bax: BCL2-associated X protein
Bcl-2: B-cell lymphoma 2 protein
CarEs: Carboxylesterases
c-Fos: c-Cellular oncogene
CML: Chronic myeloid leukemia
c-myc: MYC proto-oncogene, bHLH transcription factor
COX-2: Cyclooxygenase 2
CTD: Cantharidin
CYP2C9: Cytochrome P450 2C9
CYP2D6: Cytochrome P450 2D6
CYP3A4: Cytochrome P450 3A4
DCs: Dendritic cells
DKK3: Dickkopf WNT signaling pathway inhibitor 3
DR4/5: Death receptor 4/5
ECM: Extracellular matrix
ERCC: DNA excision repair protein
ERK: Extracellular signal-regulated kinases
ERS: Endoplasmic reticulum stress
FANCG: Fanconi anemia group G protein
GC/MS: Gas chromatography-mass spectrometry
GLUT1: Glucose transporter 1
GSTs: Glutathione S-transferases
GTP: Guanosine triphosphate
HIF-1: Hypoxia-inducible factor-1
JNK: c-Jun N-terminal kinase
MAPK: Mitogen-activated protein kinase
MC: Molluscum Contagiosum
MCF-7: Michigan Cancer Foundation - 7
MMP: Matrix metalloproteinase
Myh7: Myosin heavy chain 7
NCTD: Norcantharidin
NF-κB: Nuclear factor kappa-B
P450s: Cytochrome P450 monooxygenases
PERK/CHOP: Protein kinase R-like ER kinase/C/EBP-homologous protein
PI3K: Phosphatidylinositol 3-kinase
PKC: Protein kinase C
PLD2: Phospholipase D2
PP1: Protein Phosphatase 1
PP2A: Protein phosphatase 2A
ROS: Reactive oxygen species
TLR4: Toll-like receptor 4
TNC: Tenascin C
TNF-α: Tumor necrosis factor-α
TRAIL: Tumor necrosis factor-related apoptosis-inducing ligand
UPLC-Q-TOF/MS: Ultra performance liquid chromatography-quadrupole-time-of-flight mass spectrometry
VC: Vitamin C
VEGF: Vascular endothelial growth factor
β-TrCP: beta-transducin repeats-containing protein
